# Comparison of several methods for pain management after video-assisted thoracic surgery for pneumothorax: an observational study

**DOI:** 10.1186/s12871-019-0797-4

**Published:** 2019-07-06

**Authors:** Pierre-Antoine Allain, Michele Carella, Apostolos C. Agrafiotis, Julien Burey, Jalal Assouad, El-Mahdi Hafiani, Yacine Ynineb, Francis Bonnet, Marc Garnier, Christophe Quesnel

**Affiliations:** 1 Service d’Anesthésie-Réanimation et Médecine Péri-opératoire, AP-HP Hôpital Tenon, 4 rue de la Chine, 75020 Paris, France; 2 Service de chirurgie thoracique, AP-HP Hôpital Tenon, 4 rue de la Chine, 75020 Paris, France; 3 Faculté de médecine Sorbonne Université (Paris VI), Paris, France

**Keywords:** Video-assisted thoracic surgery, Pneumothorax, Postoperative pain, Serratus plane block, Paravertebral block

## Abstract

**Background:**

There is no defined gold standard for pain management after video-assisted thoracic surgery (VATS) for pneumothorax. In addition to systemic analgesia, various loco-regional analgesic techniques have been proposed but remain poorly evaluated in this context. We aimed to assess the analgesic efficacy of several of these techniques for the management of postoperative pain.

**Methods:**

We conducted a monocentric prospective observational cohort study from February 2017 to April 2018 in patients suffering from spontaneous pneumothorax and scheduled for VATS (*n* = 59). Patients received systemic analgesia (i) alone (*n* = 15); (ii) combined with a continuous paravertebral block (*n* = 9); (iii) combined with a continuous serratus plane block (*n* = 19); or (iv) single-shot serratus plane block (*n* = 16) as decided by the attending physician. Pain scores and analgesic-related side effects were prospectively collected by an independent observer during the first postoperative 72 h. The primary endpoint criterion was the cumulative oral morphine consumption at the end of the third postoperative day. Statistical analysis used univariate and multivariate step-by-step forward logistic regression models to determine risk factors associated with the main criteria.

**Results:**

Mean pain scores and morphine consumption were not significantly different between the 4 groups. In the multivariate analysis, the use of a continuous serratus plane block through a catheter was the only technique associated with a reduced incidence of high-dose oral morphine consumption (OR 0.09–95%CI [0.01–0.79], *p* = 0.03).

**Conclusion:**

This study suggests that serratus plane block combined with continuous infusion through a catheter may have some benefits, although further studies are needed to confirm these results and determine the true place of the serratus plane block in pain management after VATS for pneumothorax.

## Background

Spontaneous pneumothorax (SP) has an overall incidence of 24/100,000 in caucasian males [1]. Recurent and/or complete pneumothorax, compromising normal breathing and oxygenation, requires chest drainage and prevention of further recurrences. Thus, surgical treatment of pneumothorax is indicated in cases of SP relapse, bilateral pneumothorax and persistent air leak. Among the surgical procedures performed to avoid pneumothorax recurrence, the most common is pleurodesis by mechanical abrasion of the parietal pleura. The second most common is pleurectomy followed by chemical pleurodesis by intrathoracic instillation of talc [2]. These procedures are performed either through video-assisted thoracic surgery (VATS) or thoracotomy. Although VATS is associated with less intense postoperative pain [3], it remains a painful surgical procedure. Indeed, a moderate to intense postoperative pain is reported during the first postoperative 72 h and especially within the first 24 h [4]. The use of loco-regional analgesia is recommended to control postoperative pain after VATS as it allows morphine sparing and facilitates early postoperative rehabilitation [5]. Different loco-regional analgesic techniques could be used to control pain after pneumothorax surgery such as a paravertebral block, an intercostal block, an intrapleural block or more recently a serratus plane block [5]. However, some of them have limitations such as the requirement for multiple injections in the case of an intercostal block. In addition, when a pleurectomy is performed, the loss of the parietal lining of the pleura decreases the efficacy of a paravertebral block [6]. Moreover, the comparative performances of these techniques have been poorly evaluated. Thus, in current practice, there is no gold standard analgesic strategy for pneumothorax surgery.

Every year, more than one hundred patients are referred to the on-call emergency unit for pneumothorax management in our institution, among whom 60% require surgical treatment. Postoperative analgesia is commonly ensured by a loco-regional analgesic technique, but the choice of technique remains at the discretion of the attending anesthesiologist. Consequently, we evaluated the efficacy and the side effects of several techniques in a cohort of patients scheduled for VATS to treat SP.

## Methods

This study followed the STROBE statements [7]*.*

### Patient selection

This study is a monocentric prospective observational cohort study conducted at Tenon Hospital, Paris, France. Consecutive patients over 18 years old and suffering from pneumothorax requiring VATS treatment between February 2017 and April 2018 were included in the study. The exclusion criteria were: secondary pneumothorax, pregnancy, chronic preoperative analgesic treatment and opioid addiction, associated hemothorax, supra-segmental parenchymal resection and the lack of social health insurance coverage. Intraoperative conversions of VATS into thoracotomy and revision surgery within the first postoperative 72 h were also excluded from the study.

### Anesthesia, surgery and analgesia management

Except for the choice of the postoperative loco-regional analgesic technique, the anesthetic management of patients undergoing VATS for pneumothorax is standardized in our institution. All the patients were anesthetized using a targeted concentration infusion of propofol (Fresenius Kabi; Bad Homburg vor der Höhe, Germany) and sufentanil (Mylan; Canonsburg, PA, USA), while the depth of anesthesia was monitored with a bispectral index coupled with standard anesthetic monitoring. Atracurium (Hospira; Lake Forest, IL, USA) was used for muscle relaxation. Dexamethasone 8 mg IV (Mylan) was systematically used to reduce postoperative nausea and vomiting. Intraoperative ketamine administration (0.3 mg/kg – Panpharma; Boulogne-Billancourt, France) was considered as a co-analgesic except in cases of high blood pressure in the operating room, or significant active cannabis intoxication. Oral postoperative multimodal analgesia routinely included acetaminophen (1 g 4 times a day – Sanofi-Aventis, Paris, France), nefopam (20 mg 4 times a day – Biocodex; Gentilly, France) and tramadol (50 mg 4 times a day – Meda Pharma, Solna, Sweden). Intraoperative IV lidocaine (0.3 to 0.5 mg/kg – Aguettant; Lyon, France) and postoperative ketoprofen (50 mg 4 times a day – Sanofi-Aventis) administrations were left to the discretion of the attending anesthesiologist. All patients were immediately extubated at the end of the surgical procedure.

Surgery was conducted under one-lung ventilation after intubation with a double-lumen tube. Single-port VATS was the first choice method; multi-port surgery being reserved for cases when that failed. VATS was standardized as previously described [8], with a 1.5–2 cm single incision performed on the axillary anterior line in the seventh intercostal space. Wound retractors were used to protect intercostal tissues. All the instruments were introduced through this single port. Surgery consisted of resection of apical parenchymal dystrophies using an articulated stapler, followed by a talc effusion pleurodesis. A single 24F chest tube was introduced through the single incision under visual control at the end of the procedure.

The loco-regional analgesic technique was left to the discretion of the physician in charge of the patient and was performed at the end of the surgical procedure. A paravertebral block was performed by the surgeon through a transparietal approach with insertion of a catheter under direct vision until placement of the tip into the paravertebral space [9], and then injected with an initial bolus of 15 mL lidocaine 20 mg/mL + epinephrine 50 μg/mL (Aguettant), followed by a continuous injection of 10 mL/h ropivacaine 2 mg/mL (Fresenius Kabi) through an elastomeric pump. A serratus plane block was performed by the injection of local anesthetics between the serratus anterior and the latissimus dorsi muscles, with ultrasound guidance, as described initially by Blanco et al. [10]. A serratus plane block was performed either by single injection (ropivacaine 2 mg/mL, 20 mL for patients under 175 cm and 30 mL for patients taller than 175 cm) or by injection of an initial bolus (lidocaine 20 mg/mL + epinephrine 50 μg/mL, 15 mL) followed by the placement of a catheter between the two muscles and the continuous injection of 10 mL/h ropivacaine 2 mg/mL through an elastomeric pump. In addition, 10 mg on-demand oral rapid-release morphine (Actiskenan®, Ethypharm, Saint-Cloud, France) was given to patients in case of residual pain (Visual Analog Scale (VAS) value > 3/10). We predefined a threshold of 50 mg of morphine over the first 3 postoperative days as being “high-dose morphine consumption”, based on the mean amount of morphine consumption observed in our institution and the limited data available from recent studies reporting postoperative morphine consumptions in patients scheduled for VATS [11, 12].

### Data collection

We collected demographic data (age, sex, weight, height, body mass index), as well as information about active smoking status and chronic drugs consumption (especially cannabinoids and opioids) for each patient. The following surgical data were also collected: the number, side and site of surgical incisions and thoracic drains, the duration of surgery, the surgical technique used (pleurodesis or pleural abrasion, with or without resection of dystrophic parenchyma) and the duration of postoperative drainage. We also recorded the anesthetic and analgesic agents administered to patients during anesthesia, such as ketamine, intravenous lidocaine, dexamethasone, paracetamol, nefopam, tramadol, ketoprofen and/or morphine.

The morphine titration cumulative dose and pain intensity score (graded from 0 to 10 on a VAS) at rest and on coughing were collected in the recovery room upon the patient’s arrival and after one hour. Finally, pain VAS values were also collected at rest and on coughing in the wards at 2, 12, 24, 48 and 72 h after surgery. Cumulative oral morphine consumption was collected as well as daily and total doses of other analgesics such as acetaminophen, nefopam, tramadol, IV lidocaine and ketoprofen. The occurrence of nausea, vomiting and urinary retention were noted for each patient. The hospital length-of-stay was recorded.

### Definition of endpoints and statistical analysis

The primary endpoint was the cumulative oral morphine consumption (expressed in mg) during the first postoperative 72 h. The secondary endpoints were: 1) the intensity of postoperative pain at rest and on coughing, and 2) the incidence of side effects most frequently associated with opioid consumption such as nausea, vomiting and urinary retention. Quantitative data were expressed as median values [25-75th percentiles] and qualitative data were expressed as numbers (percentages). Primary and secondary endpoints for each analgesic technique used were compared using the Kruskall-Wallis test with Dunn’s correction. We defined a priori a morphine consumption > 50 mg within the first postoperative 72 h as a reasonable marker of difficult pain control and high-dose morphine consumption. Factors associated with a high-dose morphine consumption were assessed by univariate logistic regression and chi-square for quantitative and qualitative variables, respectively. All variables with *p* value ≤0.2 were integrated into the multivariate step by step forward logistic regression model. *P* < 0.05 was considered significant. Statistical analysis was performed using SPSS version 23 (SPSS, IBM Corp, Armonk, NY).

## Results

**Fig. 1:**
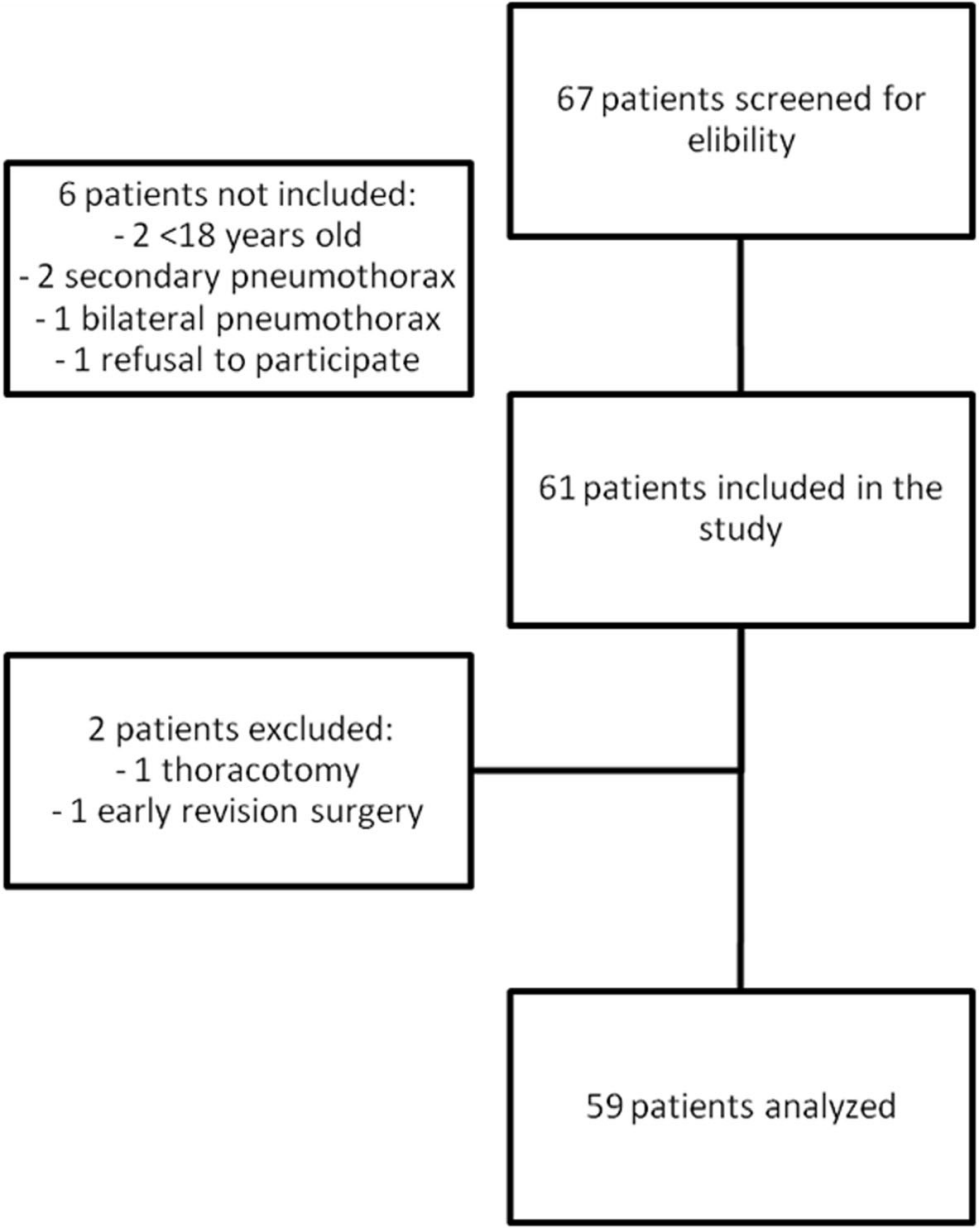
Flow diagram of the study

Sixty-seven patients underwent VATS for SP during the study period, out of whom 61 were included in the study (Fig. 1). Among the 6 patients who were not included, 2 were under 18 years of age, 2 had pneumothorax secondary to trauma and bullous emphysema, 1 had bilateral pneumothorax and 1 refused to participate. Additionally, 2 more patients were excluded due to intraoperative conversion to thoracotomy and early reoperation for hemorrhage, respectively (Fig. 1). The demographics of the patients and the surgical procedures are described in Table 1. Among the 59 patients analyzed, 10 (17%) had a past history of lung disease and 17 (28%) had recurrent SP. A standardized single port procedure was performed in 92% of the cases. The median operative time was 75 [60–100] minutes.

**Table 1:** Patients’ characteristics

Patient’s characteristics (*n* = 59)	n (%) or median [25^e^-75^e^ percentiles]
Preoperative characteristics, *n (%) or median [IQR]*	
Gender (male)	44 (75%)
Age (years)	34 [26–42]
BMI (kg.m^−2^)	20.9 [18.9–23.0]
ASA I class	40 (68%)
Respiratory comorbidity	10 (17%)
Past history of pneumothorax	17 (29%)
Past history or active smoking	40 (68%)
Cannabis consumption	19 (32%)
Hypertension	5 (9%)
Surgical procedure, *n (%) or median [IQR]*	
One-port VATS	54 (92%)
Chemical pleurodesis	46 (78%)
Bullous/wedge resection	36 (61%)
Duration of surgery (min)	75 [60–100]
Single chest tube	54 (92%)
Duration of postoperative thoracic drainage (days)	4 [3.0–5.0]
Recovery room, *n (%) or median [IQR]*	
Extubation immediately after surgery	59 (100)
Dose of morphine titration	10 [5.5–14.0]

Sixteen patients (27%) had a serratus plane block with a single injection and 19 (32%) had a serratus plane block with insertion of a catheter for continuous loco-regional analgesic infusion. A paravertebral block with insertion of a catheter was performed in 9 patients (15%). Fifteen (25%) patients received oral multimodal analgesia alone, including rapid-release morphine. Values of pain intensity score corresponding to the analgesic technique used are reported in Table 2.

**Table 2:** Postoperative Visual Analog Scale values and incidence of postoperative nausea and vomiting and urinary retention according to the analgesic protocol

Visual Analog Scale values								PONV	Urinary retention
time	H0	H1	H2	H12	H24	H48	H72		
Systemic analgesia alone *(n = 15)*									
At rest	4.5 [2–7]	4 [4–6]	3 [2–4]	3 [2–6]	3 [2–6]	2 [1.5–4]	2 [0–4]	3 (21%)	1 (7%)
On coughing	6.5 [5–8]	7 [5–8]	5 [4–6]	5 [4–7]	5 [4–7]	5 [4–5]	3 [2.5–5]		
Paravertebral block with continuous infusion through a catheter *(n = 9****)***									
At rest	5 [1–6]	6 [1–6]	4 [1–4]	4 [2–5]	3 [2–4]	2 [2–4]	2 [0–4]	2 (22%)	2 (22%)
On coughing	8 [4.5–9]	8 [4.5–9]	4 [3.5–5.5]	6 [4–9.5]	4.5 [3–6]	5 [3–7.5]	4 [2.5–5]		
Single-injection serratus plane block *(n = 16)*									
At rest	4.5 [0.5–7]	5.5 [3–6]	3 [1–3.5]	3 [3–4]	3.0 [3–4]	3 [2–4]	2 [0–3]	2 (13%)	1 (6%)
On coughing	6.5 [1–8]	6 [3–7]	4 [2–5]	5 [4–7.5]	4.5 [3–6]	5 [4–6]	3 [2–4]		
Serratus plane block with continuous infusion through a catheter *(n = 19)*									
At rest	4.5 [0–6]	3 [0–6]	1 [0–3]	3 [2–5.5]	2 [1.5–4.5]	2 [0–4]	1 [0–2]	8 (42%)	0 (0%)
On coughing	6 [1–8]	5 [3–7]	3 [3–5]	4.5 [3–6]	3 [3–6]	4 [1–5]	2 [2–3]		

Values are expressed as median [25^e^-75^e^ percentiles] or n (%)

*PONV* postoperative nausea and vomiting

**Fig. 2:**
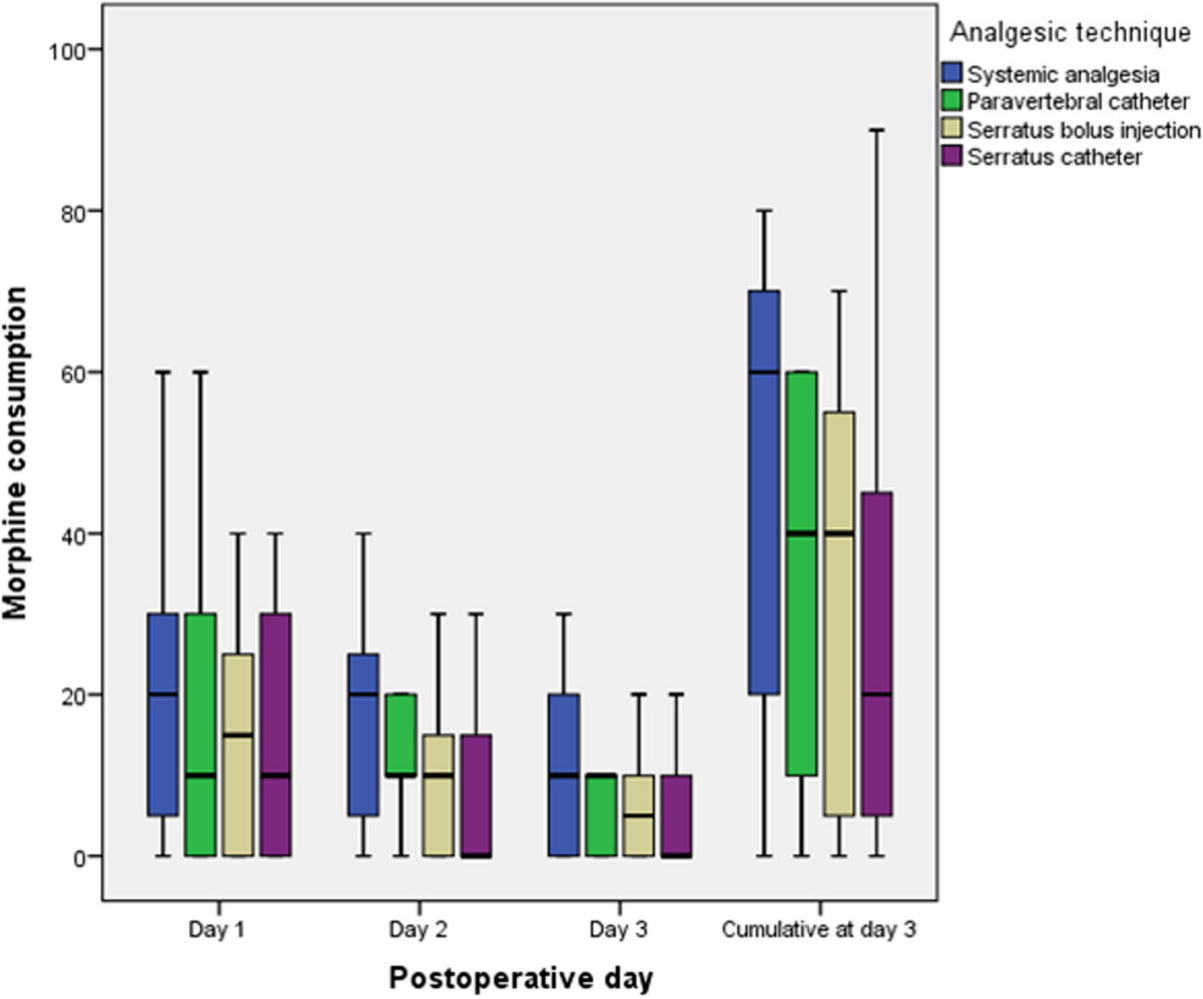
Daily and cumulative oral morphine consumption during the first postoperative 72 h according to the postoperative analgesic technique used

Morphine consumptions are reported in Fig. 2. Morphine consumption and VAS values were not different for the 4 groups (*p* > 0.05 - Table 3 & Fig. 2). There were no significant differences in the incidence rates of postoperative nausea, vomiting or urinary retention across the groups. The cumulative postoperative oral morphine consumption at 72 h was 30 [10–60] mg. Over the study-period, 19 (32%) patients required > 50 mg of oral morphine to control their pain. In the univariate analysis, parameters with a *p* value ≤0.2 when comparing patients with low-dose and high-dose morphine consumption were the use of a continuous serratus plane block (*p* = 0.06), the use of systemic analgesia alone (*p* = 0.02), a single-port incision (*p* = 0.16) and the postoperative use of tramadol as a co-analgesic (*p* = 0.19) (Table 3). In the multivariate analysis, the continuous serratus plane block was the only variable independently associated with reduced high-dose morphine consumption (OR 0.09, 95%CI [0.01–0.79]; *p* = 0.03) (Table 4). Non-significant association were found between reduced high-dose morphine consumption and the postoperative use of tramadol (*p* = 0.057) and a single-port surgical procedure (*p* = 0.052) (Table 4).

**Table 3:** Univariate analysis of parameters in patients with cumulative oral morphine consumption ≤50 mg and > 50 mg during the first postoperative 72 h

Cumulative morphine at D3	≤ 50 mg (*n* = 40)	> 50 mg (*n* = 19)	P
Age > 40 years old	11 (28%)	7 (36.8)	0.46
Gender (male)	31 (78%)	13 (68.4)	0.45
ASA class > I	14 (35%)	5 (26.3)	0.50
BMI (kg.m^−2^)	21 [19–23]	21 [18–22]	0.88
Preoperative chest tube > 24 h	26 (65%)	15 (79%)	0.27
Hypertension	4 (10%)	1 (5%)	0.54
Diabetes mellitus	1 (3%)	0 (0%)	0.48
Respiratory comorbidity			0.65
Asthma	3 (8%)	1 (5%)	
COPD	3 (8%)	0 (0%)	
Interstitial lung disease	1 (3%)	0 (0%)	
Emphysema	1 (3%)	1 (5%)	
Past history of pneumothorax	11 (28%)	6 (32%)	0.74
Cannabis chronic use	12 (30%)	7 (37%)	0.59
Single-port incision	38 (95%)	16 (84%)	0.16
Chemical pleurodesis	32 (80%)	14 (74%)	0.58
Pulmonary resection	23 (58%)	13 (68%)	0.42
Postoperative chest tube duration > 1 day	4 (10%)	1 (5%)	0.54
Duration of surgery > 60 min	21 (53%)	13 (68%)	0.24
Anesthetic and analgesic agents			
Total dose of sufentanil used *(μg/kg)*	0.89 [0.69–1.11]	0.81 [0.71–1.22]	0.76
Ketamine	31 (78%)	15 (79%)	0.90
Intravenous Lidocaine	26 (65%)	11 (58%)	0.59
Dexamethasone	38 (95%)	17 (90%)	0.43
Acetaminophen	40 (100%)	19 (100%)	–
Nefopam	39 (98%)	18 (95%)	0.58
Ketoprofen	22 (55%)	11 (58%)	0.83
Tramadol	39 (98%)	17 (90%)	0.19
Loco-regional analgesic technique			
Systemic analgesia alone	6 (15%)	9 (47%)	0.02
Paravertebral catheter	6 (15%)	3 (16%)	0.93
Serratus single injection	12 (30%)	4 (21%)	0.47
Serratus catheter	16 (40%)	3 (16%)	0.06

Values are expressed as median [25^e^-75^e^ percentiles] or n (%)

COPD: chronic obstructive pulmonary disease

**Table 4:** Multivariate analysis of factors associated with high-dose cumulative oral morphine consumption during the first postoperative 72 h

Variables	Odd ratio [95%CI]	P
Postoperative tramadol use	0.04 [0.002–1.09]	0.057
Serratus catheter	0.09 [0.01–0.79]	0.03
Single-port surgery	0.07 [0.005–1.01]	0.052

## Discussion

This prospective cohort study shows relatively high pain intensity and morphine consumption after VATS for pneumothorax. In this context, the serratus plane block with insertion of a catheter for continuous local anesthetic infusion, in addition to multimodal systemic analgesia, provided satisfactory pain control during the first postoperative 72 h and decreased high-dose morphine consumption.

Pain treatment after thoracic surgery is challenging, thoracotomy being considered as one of the most painful surgical procedures. The gold standard for pain treatment after thoracotomy is epidural analgesia [13]. However, there is evidence to suggest that a paravertebral block could be equally effective with less side effets [13–15]. As thoracic surgery patients take part in enhanced rehabilitation strategies that require capability to deambulate, and aim to achieve rapid recovery, side effects induced by epidural analgesia, such as orthostatic hypotension and urinary retention, are especially deleterious and may preclude the application of such a postoperative rehabilitation program. That said, a paravertebral block could be less efficient after pleurodesis due to pleural inflammation or surgical dilaceration of the parietal lining of the pleura [6]. This may explain the trend towards higher pain scores in patients having undergone a paravertebral block in our study, and could make the use of a paravertebral block unsuitable for ensuring analgesia in this context.

VATS is considered less painful than thoracotomy [3], explaining why anesthesiologists consider epidural analgesia less appropriate [16]. Nevertheless, loco-regional analgesic techniques are still included routinely in postoperative analgesic protocols to provide good quality analgesia in VATS patients [5]. The choice of the appropriate loco-regional analgesic technique is determined by the extent and duration of the pain. In the context of pneumothorax surgery, the duration of pain is mainly related to the duration of postoperative chest drainage. In this cohort of patients, despite the fact that all loco-regional analgesic techniques resulted in satisfactory pain control after a couple of hours, the serratus plane block was the only technique that reduced the number of patients requiring high-dose oral morphine as an analgesic rescue treatment. The serratus plane block, as described by Blanco et al. was initially introduced in patients scheduled for breast surgery [10]. In a previous study, Okmen et al. reported that performing a single-bolus serratus plane block resulted in lower pain intensity and smaller quantities of tramadol being administered as intravenous patient-controlled analgesia (PCA) after VATS [17]. However, the control group that only received IV tramadol PCA was far from the standard of care for this procedure, and the study was restricted to the first postoperative 24 h while postoperative pain is supposed to last over a longer time period. Park et al. documented that a single-injection serratus plane block performed before surgery decreased both intraoperative remifentanil requirements and postoperative fentanyl consumption compared with a sham block [18]. In another study using a comparable design, Kim et al. documented lower pain scores, reduced morphine consumption and a higher degree of satisfaction in patients treated with a serratus plane block compared to a sham block [19]. Although properly designed and showing the efficacy of the serratus plane block, these two prospective studies, as well as the Okmen et al. study, did not compare the serratus plane block to a standard of care considered appropriate for this kind of surgical procedure. Kalil et al. have compared thoracic epidural anesthesia to serratus plane block with insertion of a catheter and documented no difference in pain scores in a limited number of patients scheduled for VATS [20]. Conversely, in our study, the serratus plane block was associated with an opioid sparing effect that was not found for the other loco-regional analgesic techniques. In addition, the fact that continuous infusion through a catheter was associated with reduced high-dose morphine consumption over the first postoperative 3 days, unlike a single-injection serratus plane block, supports the placement of such a catheter to maintain an effective postoperative analgesia over several days. Taken altogether, these data suggest that the serratus plane block may be a technique to ensure good pain relief and morphine sparing after VATS for pneumothorax.

This study has several limitations. First of all, the number of patients included in the study is limited making it only a preliminary assesment of the effect of a serratus plane block. Indeed, although the serratus plane block with continuous infusion through a catheter is associated with reduced high-dose morphine consumption, we did not observe any significant difference in the global morphine consumption between the different groups. Although a serratus plane block is theoretically advantageous in the context of pleurodesis, we did not report any superiority of the serratus plane block over the paravertebral block regarding pain score or global morphine consumption. However, it could be hypothesized that our study lacks power to demonstrate such a superiority, in particular as a trend towards a lower morphine consumption in the continuous serratus plane block group existed. Further larger studies are required to confirm these preliminary results.

Secondly, since it is not a double-blind randomized study, several biases may have been introduced. However, pain scores and morphine administration were not evaluated by the physicians who performed the block in the operating theatre, providing an acceptable assessment of postoperative pain and morphine consumption. However, it can not be ruled out that certain differences in intra-operative features (such as duration of the surgery, actual doses of anesthesics and analgesics delivered, use of a non-steroidal anti-inflammatory drug that was left to the discretion of the attending anesthesiologist, etc.) may have impacted the pain scores and postoperative morphine consumptions, which could only have been avoided with a randomized design. It could also be hypothesized that, in the absence of randomization, surgical features may have impacted both postoperative pain scores and locoregional analgesia efficacy. However, 92% of patients had single-port VATS, which is a standardized procedure in our structure [8], thus limiting the risk of non-balanced effects of the surgery between groups.

Thirdly, the definition of the morphine consumption threshold used in our study could be challenged. We chose a threshold of 50 mg of cumulative oral morphine administration over the first postoperative 72 h to define high-dose consumption and assess the efficacy of the loco-regional analgesic techniques. As there is no agreed definition of what constitutes high-dose morphine consumption after VATS for pneumothorax, we chose the mean value from our institution. It should be noted that the validity of this threshold is reinforced by other recent studies reporting close postoperative morphine consumptions in patients scheduled for VATS [11, 12]. The clinical relevance of the opoid sparing effect of serratus plane block could also be challenged as the multivariate analysis suggests a link between single-port surgery and a reduced need for postoperative high-dose morphine; a finding in line with a previous retrospective study [4]. However, most of the patients of this cohort had single port surgery as it has been reported to be a safe and less invasive technique for SP surgery [8], suggesting that even with an optimized surgical technique a serratus plane block with catheter remains a valid choice for postoperative pain management. Along the same lines, tramadol administration tended to reduce the need for high-dose morphine. Using more tramadol postoperatively resulted in fewer patients using morphine on demand. Tramadol administation also explains why the incidence in postoperative nausea and vomiting was comparable in all the groups independently of morphine consumption.

## Conclusions

This study suggests that serratus plane block combined with continuous infusion through placement of a catheter may have some benefits, although further studies are needed to confirm these results and determine the true place of the serratus plane block in pain management after VATS for pneumothorax.

## Data Availability

The datasets used and analysed during the current study are available from the corresponding author on reasonable request.

## Abbreviations

PCA: Patient-Controlled Analgesia
SP: Spontaneous Pneumothorax
VAS: Visual Analog Scale
VATS: Video-Assisted Thoracic Surgery
